# Supplementary material for: Asymmetric Push–Pull Type Co(II) Porphyrin for Enhanced Electrocatalytic CO2 Reduction Activity
Source: Molecules. 2022 Dec 24;28(1):150. doi: 10.3390/molecules28010150 (PMC9822202; doi:10.3390/molecules28010150)
Supplement: Supplementary file 1 [file molecules-28-00150-s001.zip › molecules-2100848-supplementary.pdf]

Supplementary Materials

FOR

Asymmetric Push–Pull Type Co(II) Porphyrin for Enhanced Electrocatalytic CO_2 Reduction Activity

Chenjiao Huang [1], Wenwen Bao [1], Senhe Huang [2], Bin Wang [1], Chenchen Wang [1], Sheng Han [1,*], Chenbao Lu [2,*] and Feng Qiu [1,*]

[1] School of Chemical and Environmental Engineering, Shanghai Institute of Technology, 100 Haiquan Road, Shanghai 201418, China

[2] The Meso-Entropy Matter Lab, State Key Laboratory of Metal Matrix Composites, Frontiers Science Center for Transformative Molecules, School of Chemistry and Chemical Engineering, Shanghai Jiao Tong University, 800 Dongchuan Road, Shanghai 200240, China

* Correspondence: hansheng654321@sina.com (S.H.); castle@sjtu.edu.cn (C.L.); fengqiu@sit.edu.cn (F.Q.)

1. Electrochemical measurements

The electrocatalytic CO_2 reduction was performed in a three electrode H-type cell, of which Ag/AgCl as the reference electrode, Pt plate as the counter electrode, and catalysts loaded on carbon paper as the working electrode. All potentials reported in this work were versus to reversible hydrogen electrode (RHE) using the following formula:

$$E_{RHE}\ (V) = E_{Ag/AgCl}\ (V) + 0.197\ V + 0.0591\ V \times pH$$

The electrolyte was CO_2 saturated 0.5 M $KHCO_3$, and CO_2 (Air Liquid, Ltd.) was continuously supplied to the cell (20 mL min^{-1}) through a gas bubbling tube during the constant potential electrolysis. The LSV curves were obtained with a scan rate of 5 mV s^{-1}, all potentials in this study were without *i*R compensated. The electrochemical double-layer capacitances (C_{dl}) of catalysts were calculated from CV curves. The CV curves were performed at scan rates varying from 10 to 70 mV s^{-1} in the region from -0.26 to -0.16 V. The capacitive currents of ΔJ ($J_{anodic} - J_{cathodic}$) are plotted as a function of the CV against the scan rate. The slope of the fitting line is equal to twice the C_{dl}, which is linearly proportional to the electrochemically effective surface area of the electrode. The gaseous products were monitored by an online gas chromatography (GC, Shimadzu GC-2014C), equipped with a thermal conductivity detector (TCD) detector for H_2 and a flame ionization detector (FID) detector for CO quantification. A GC run repeats every 18 minutes. The GC was calibrated with standard gas mixtures (Air Liquide, CO, H_2, CH_4, C_2H_4, C_2H_6, C_2H_2 in N_2) before the product measurements. The liquid products in the $KHCO_3$ solution were analyzed and quantified through a Bruker 500 MHZ (AVANCE Ⅲ) NMR spectroscope with water suppression. After electrolysis, $KHCO_3$ electrolyte (0.5 mL) was collected and mixed with D_2O (0.1 mL) in an NMR tube and dimethyl sulfoxide (DMSO, 0.05 μL) as an internal standard.

Faradic efficiency (FE) calculation:

Faradaic Efficiency (FE) of CO and H_2 were calculated via the following

equation:

$$\text{FE} = \frac{Q_i}{Q_{total}} = \frac{2 \times P_0 \times \mathrm{F} \times \mathrm{v} \times \nu_i}{\mathrm{R} \times \mathrm{T} \times \mathrm{I}}$$

where Q_i is the quantity of electric charge needed to produce corresponding product i. Q_{total} is the quantity of electric charge needed to produce all products. 2 is the number of electrons transferred per mole CO_2 to CO or per mole H_2O to H_2. P_o is atmospheric pressure (1.01×10^5 Pa), F is the faradaic constant (96485 C mol^{-1}), v is the gas flow rate measured by flow meter, ν_i is the volume concentration of gas product in the exhaust gas from the cell determined by online GC. T is the reaction temperature (298.15 K), R is the idea gas constant (8.314 J mol^{-1} K^{-1}), and I is the current at each potential.

Evaluation of turnover frequency (TOF) (h^{-1}):

The TOF (h^{-1}) of product CO was evaluated as follows:

$$\text{TOF} = \frac{FE_{CO} \times j_{total} \times A \times M_{Co} \times t}{n \times F \times \omega_{Co} \times m}$$

where FE is the faradaic efficiency of CO, *j* is the total current density, A (1 cm^2) is the electrode geometric area, ω_{Co} is the mass fraction of cobalt on the catalyst, m is the mass of catalyst coated on working electrode, and M_{Co} is the atomic mass of Co (59 g mol^{-1}). F is the faradaic constant (96485 C mol^{-1}), t is the reaction time (1 h/3600 s), n is the number of electrons transferred for product formation, which is 2 for CO.

DFT calculation

Electronic properties (HOMO-LUMO) of porphyrin molecules were performed using the Gaussian 09 program. A PBE0 functional with D3 correction (Becke–Johnson damping) was adopted for its robustness and dispersion corrections, which make it widely accepted as the proper functional to study the reactions of transition metal complexes. The Stuttgart–Dresden pseudopotential and double-ξ valence basis set were used for transition metal atoms (cobalt). For all other main group elements (H, C, N, O), the all-electron 6–31G* basis set was used. The geometric structures of all species were fully

optimized.

The free energy calculations were performed using the Vienna *ab* initio simulation package (VASP) with the projector-augmented wave (PAW) method. All calculations were based on the same generalized gradient approximation (GGA) method with Perdew-Burke-Ernzerhof (PBE) functional for the exchange-correlation term. The plane wave cutoff was set to 400 eV. The Brillouin zone integration was carried out with 1×1×1 Monkhorst-Pack *k*-point grid. The cells of molecules were built with vacuum slab height of 10 Å along x, y and z direction. The convergence of energy and forces were set to 5×10^{-7} eV and 0.001 eV $Å^{-1}$ for structure optimization, respectively.

The CRR pathway with CO production can be summarized as follows:

$$\text{Overall: } CO_2 + 2H^+ + 2e^- \rightarrow CO + H_2O$$

$$CO_2 + * + H^+ + e^- \rightarrow COOH^*, \qquad \Delta G_1$$

$$COOH^* + H^+ + e^- \rightarrow CO^* + H_2O, \qquad \Delta G_2$$

$$CO^* \rightarrow CO + * \qquad \Delta G_3$$

The free energy of the adsorption of intermediates including COOH* and CO* was calculated by: $G = E^{DFT} + ZPE - T\Delta S$, where E^{DFT} is the DFT-optimized total energy, ZPE is the zero-point vibrational energy, T is the temperature, and ΔS is the entropy). The zero-point energies and entropies of the reaction species were calculated from the vibrational frequencies.

2. Figures

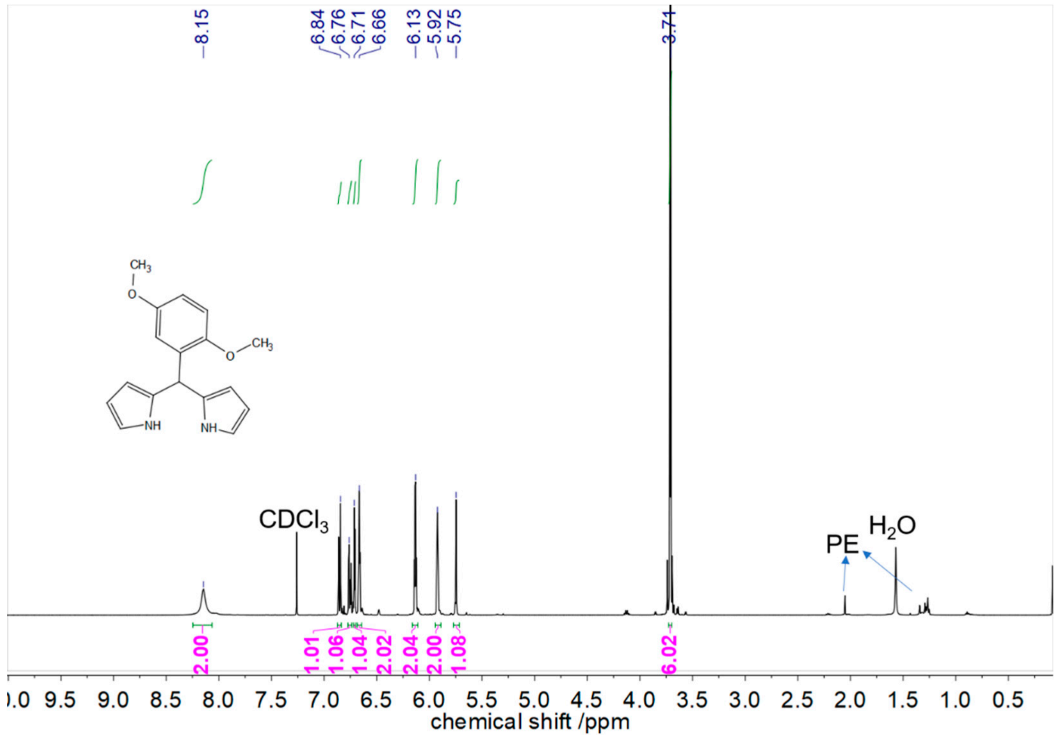

Figure S1. ^{1}H NMR spectrum of DpmPM.

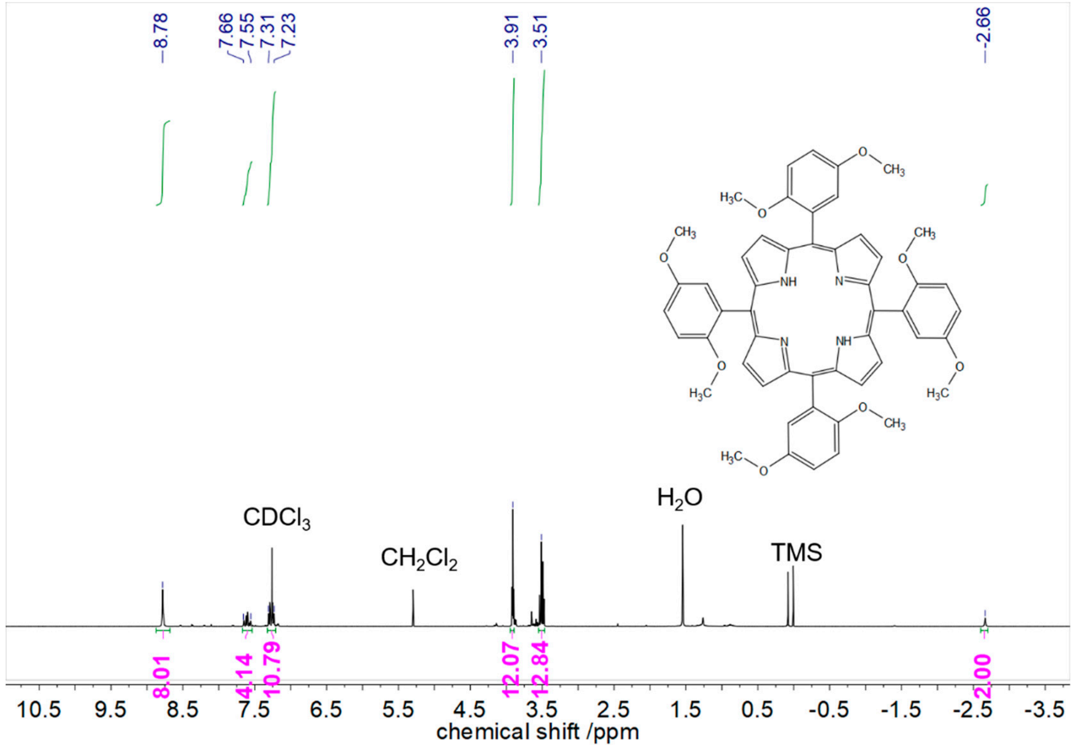

Figure S2. ^{1}H NMR spectrum of *cs*-Por-OMe.

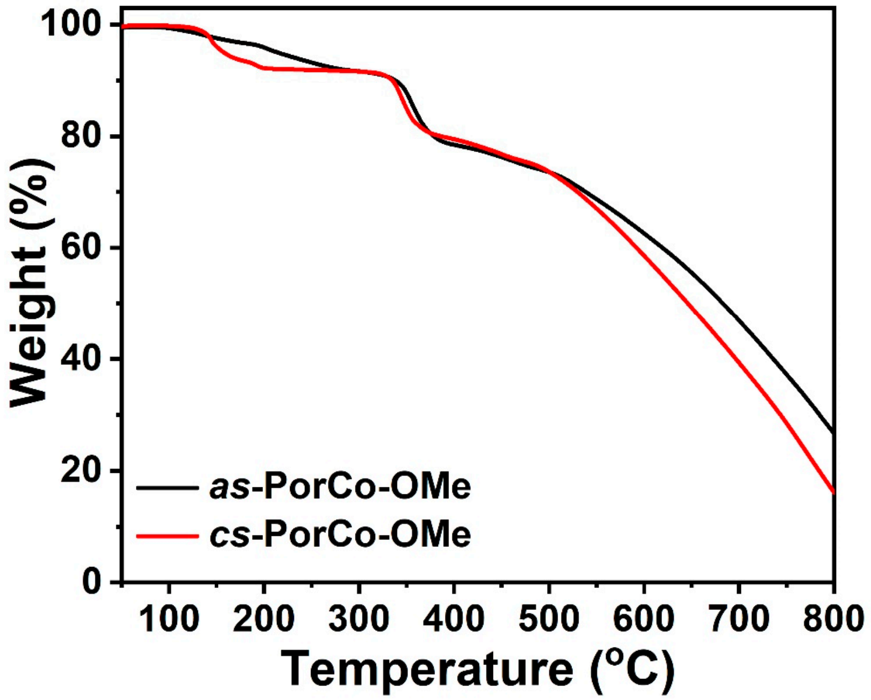

Figure S3. TGA cruves of *as*-PorCo-OMe and *cs*-PorCo-OMe

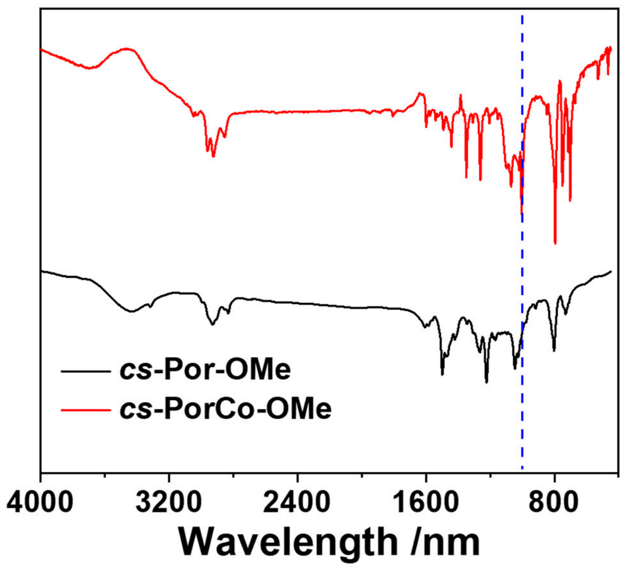

Figure S4. a) FTIR spectra of *cs*-PorCo-OMe and *cs*-Por-OMe

.

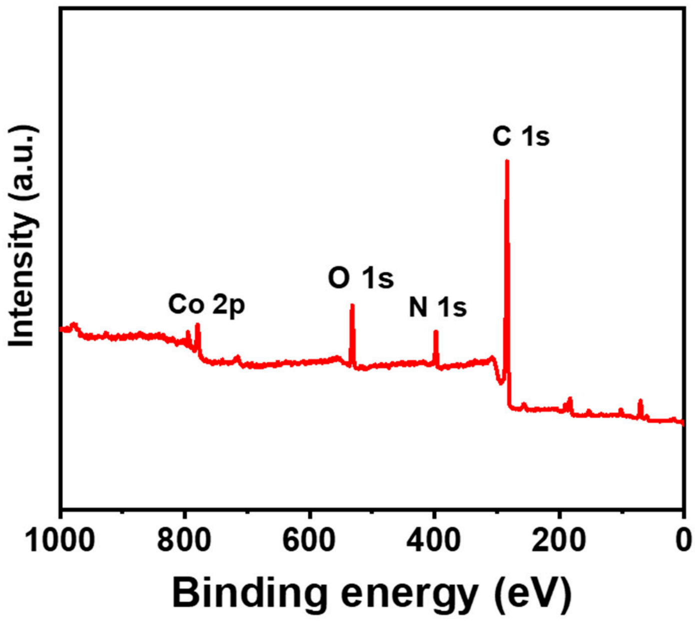

Figure S5. XPS spectra of *as*-PorCo-OMe

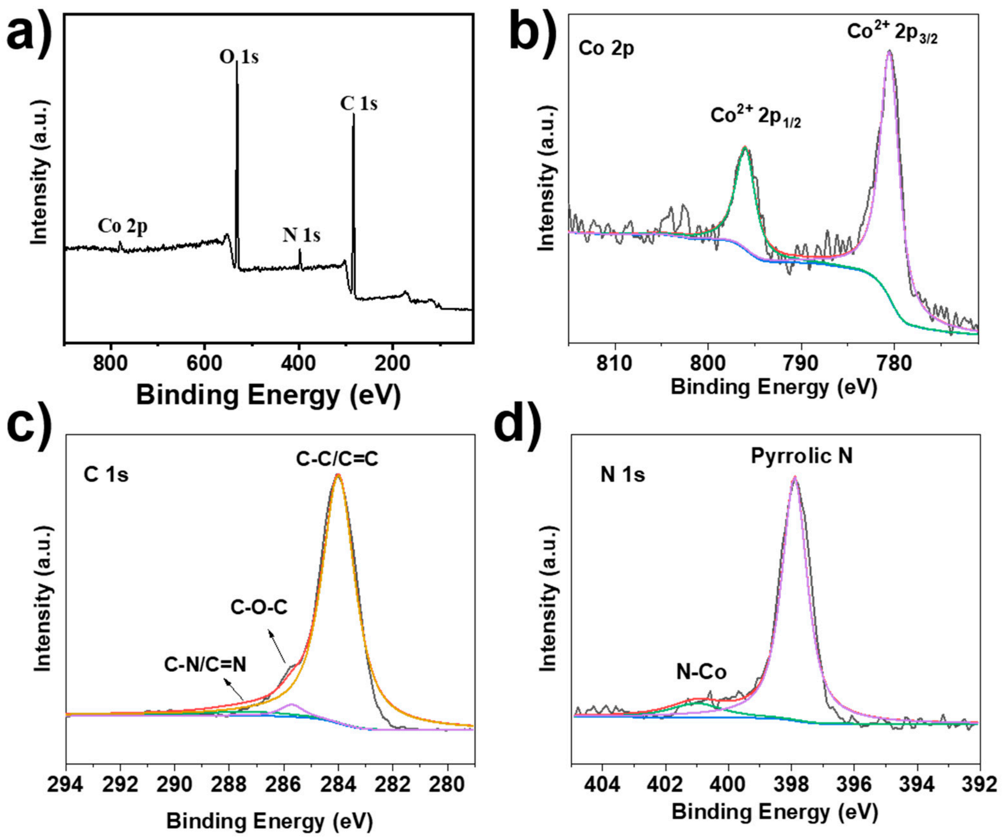

Figure S6. (a) XPS spectra of *cs*-PorCo-OMe; High resolution Co 2p (b), N 1s (c) and C 1s (d) XPS spectra in *cs*-PorCo-OMe

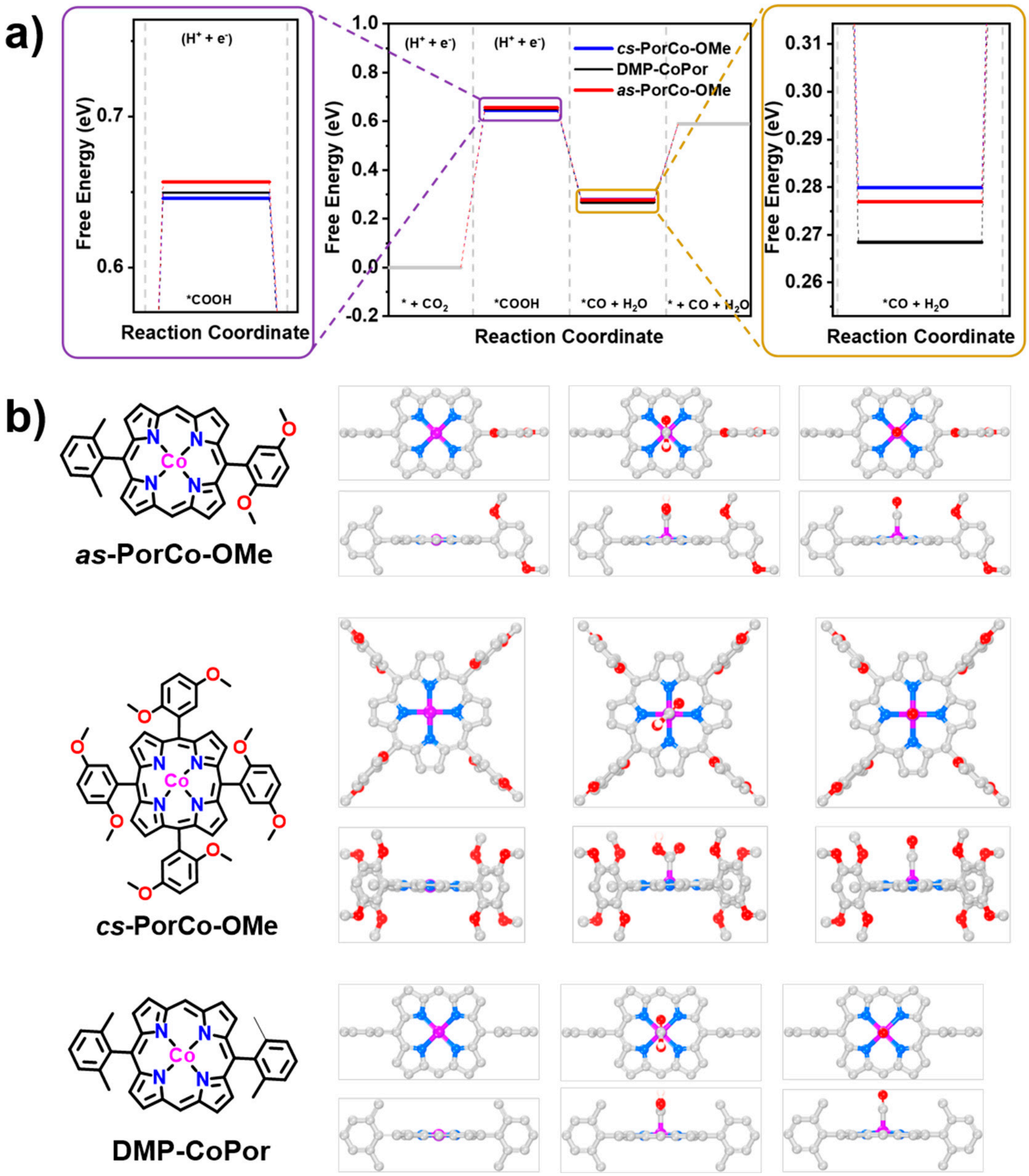

Figure S7. a) Free energy of as-PorCo-OMe and cs-PorCo-OMe in different CO2RR steps; b) Schematic of the reaction steps of CO_2 reduction to CO on *as*-PorCo-OMe, *cs*-PorCo-OMe, and DMP-CoPor.

As shown in Figure S7a, the free energy path of the conversion of CO_2 to *COOH (Δ*GCOOH) are similar for *as*-PorCo-OMe, *cs*-PorCo-OMe, and DMP-CoPor, in which *cs*-PorCo-OMe has the lowest Gibbs free energy and the free energy gap between second step and third step for *as*-PorCo-OMe (0.379 eV) also is lower than that of DMP-CoPor (0.381 eV). These results suggest that the methoxy-substitution could provide the electron donor effect on the electrocatalytic activity.

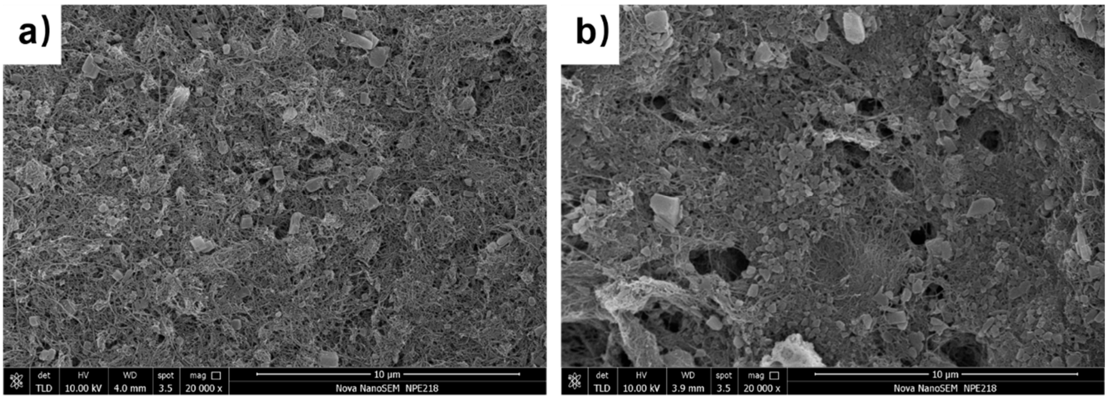

Figure S8. SEM images of *as*-PorCo-OMe (a) and *cs*-PorCo-OMe (b) with CNTs

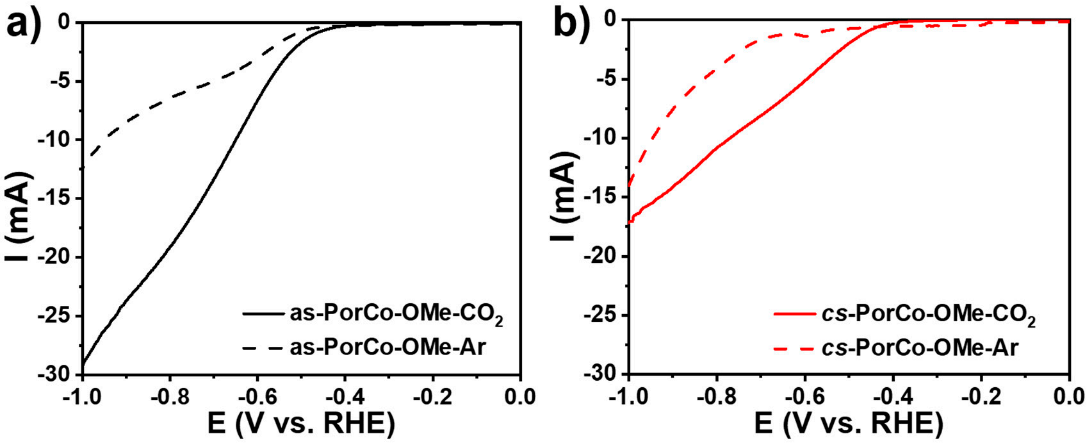

Figure S9. LSV curves in CO_2-saturated and Ar-saturated 0.5 M $KHCO_3$ electrolyte for *as*-PorCo-OMe (a) and *cs*-PorCo-OMe (b).

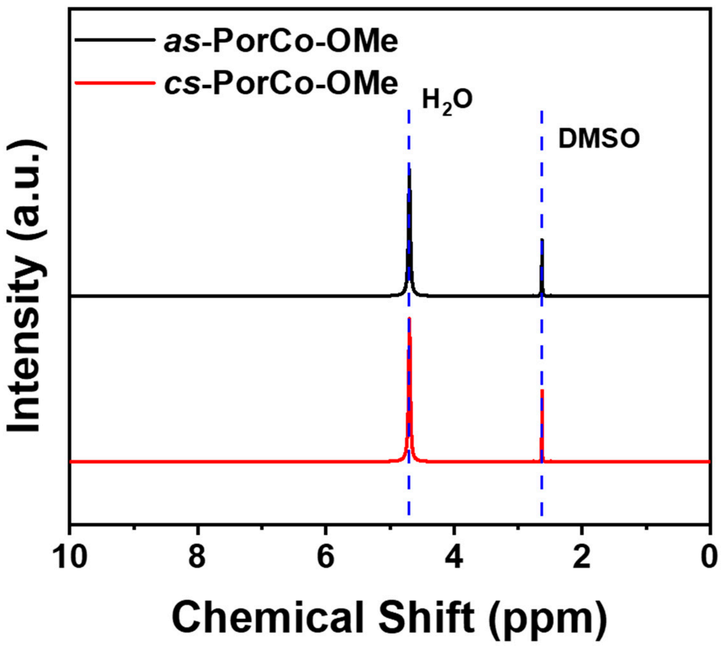

Figure S10. ¹H NMR spectra of products from electrocatalyst of *as*-PorCo-OMe (a) and *cs*-PorCo-OMe (b) at -0.7 V vs. RHE.

Except the water and solvent signal, no additional signal is detected, indicating that no liquid products formed during CO_2RR.

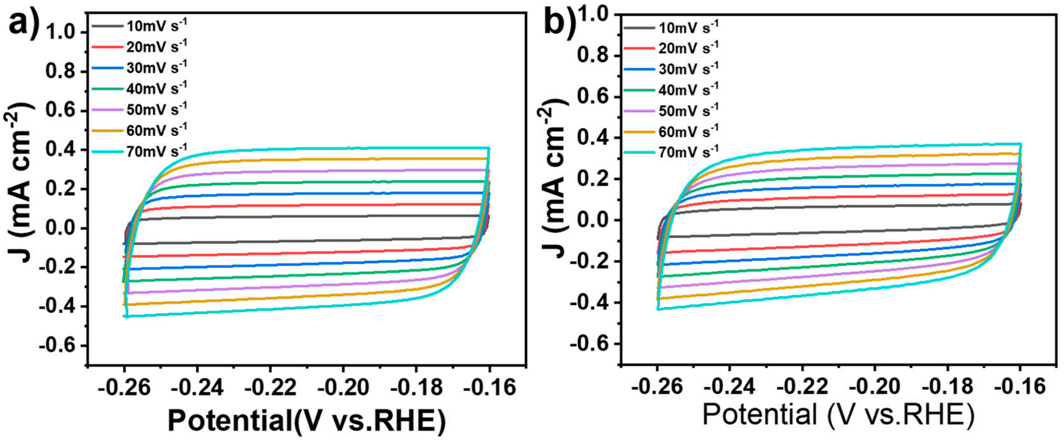

Figure S11. Cyclic voltammetry measurements were performed at scan rates varying from 10 to 70 mV s^{-1} for *as*-PorCo-OMe (a) and *cs*-PorCo-OMe (b) .

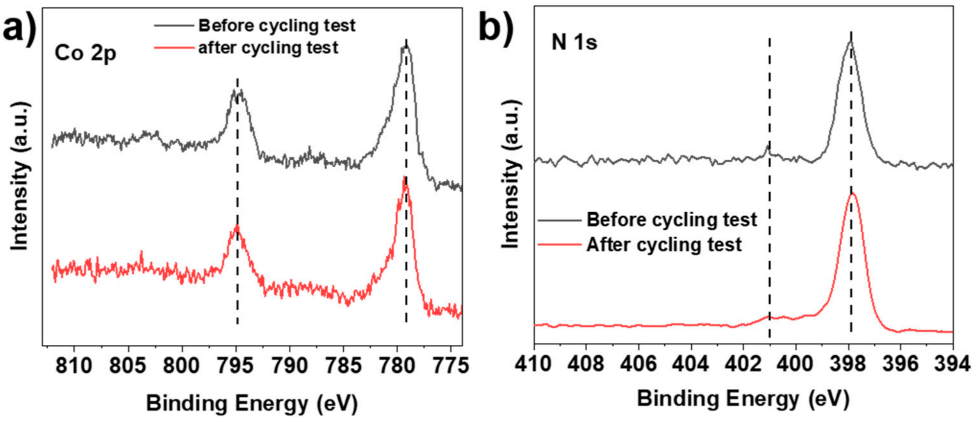

Figure S12. Co 2p (a), and N 1s (b) XPS spectra in *as*-PorCo-OMe before and after cycling test.

Table S1. Comparison of CO_2RR performance with reported electrocatalysts.

Catalysts	Electrolyte	j/mA cm^{-2}	Potential (V vs. RHE)	FE_{CO}	TOF (h^{-1})	Ref.
***as*-PorCo-OMe**	**0.5 M $KHCO_3$**	**-13.4**	**-0.7**	**94.7%**	**2880**	**This work**
***cs*-PorCo-OMe**	**0.5 M $KHCO_3$**	**-6.30**	**-0.7**	**84.5%**	**2329**	**This work**
CoTMPP	0.5 M $KHCO_3$	-0.46	-0.6	94.1%	~720	S1
as-PorCo	0.5 M $KHCO_3$	-6.25	-0.6	93%	2968	S2
DMP-CoPor	0.5 M $KHCO_3$	-4.4	-0.7	85.5%	1918	S3
Co proto-porphyrin	0.1 M $HClO_4$	-0.33	-0.7	40%	2880	S4
PorCo/cationic POP	0.5 M $KHCO_3$	~4.2	-0.6	83%	5040	S5
COF-367-PorCo(1%)	0.5 M $KHCO_3$	-0.4	-0.55	90%	764	S6
PorCo-MOF	0.1 M $KHCO_3$	-1	-0.6	76%	200	S7
PorFe-MOF	0.5 M $KHCO_3$	-1.2	-0.5	91%	1209	S8
PorNi-CTF	0.5 M $KHCO_3$	- 52.9	-0.9	97%	1692	S9

4. Reference

S1. Zhu, M.; Yang, D.-T.; Ye, R.; Zeng, J.; Corbin, N.; Manthiram, K., Inductive and electrostatic effects on cobalt porphyrins for heterogeneous electrocatalytic carbon dioxide reduction. *Catal. Sci. Technol.* **2019,** *9*, 974-980.

S2. Bao, W.; Huang, S.; Tranca, D.; Feng, B.; Qiu, F.; Rodríguez-Hernández, F.; Ke, C.; Han, S.; Zhuang, X., Molecular Engineering of Co^{II} Porphyrins with Asymmetric Architecture for Improved Electrochemical CO_2 Reduction. *ChemSusChem* **2022,** *15*, e202200090.

S3. Xuan, X.; Jiang, K.; Huang, S.; Feng, B.; Qiu, F.; Han, S.; Zhu, J.; Zhuang, X., Tertiary amine-functionalized Co(II) porphyrin to enhance the electrochemical CO2 reduction activity. *J. Mater. Sci.* **2022,** *57*, 10129-10140.

S4. Shen, J.; Kortlever, R.; Kas, R.; Birdja, Y. Y.; Diaz-Morales, O.; Kwon, Y.; Ledezma-Yanez, I.; Schouten, K. J. P.; Mul, G.; Koper, M. T. M., Electrocatalytic reduction of carbon dioxide to carbon monoxide and methane at an immobilized cobalt protoporphyrin. *Nat. Commun.* **2015,** *6*, 8177.

S5. Tang, J.-K.; Zhu, C.-Y.; Jiang, T.-W.; Wei, L.; Wang, H.; Yu, K.; Yang, C.-L.; Zhang, Y.-B.; Chen, C.; Li, Z.-T.; Zhang, D.-W.; Zhang, L.-M., Anion exchange-induced single-molecule dispersion of cobalt porphyrins in a cationic porous organic polymer for enhanced electrochemical CO_2 reduction via secondary-coordination sphere interactions. *J. Mater. Chem. A* **2020,** *8*, 18677-18686.

S6. Lin, S.; Diercks, C. S.; Zhang, Y.-B.; Kornienko, N.; Nichols, E. M.; Zhao, Y.; Paris, A. R.; Kim, D.; Yang, P.; Yaghi, O. M.; Chang, C. J., Covalent organic frameworks comprising cobalt porphyrins for catalytic CO_2 reduction in water. *Science* **2015,** *349*, 1208-1213.

S7. Kornienko, N.; Zhao, Y.; Kley, C. S.; Zhu, C.; Kim, D.; Lin, S.; Chang, C. J.; Yaghi, O. M.; Yang, P., Metal–Organic Frameworks for Electrocatalytic Reduction of Carbon Dioxide. *J. Am. Chem. Soc.* **2015,** *137*, 14129-14135.

S8. Dong, B.-X.; Qian, S.-L.; Bu, F.-Y.; Wu, Y.-C.; Feng, L.-G.; Teng, Y.-L.; Liu, W.-L.; Li, Z.-W., Electrochemical Reduction of CO_2 to CO by a Heterogeneous Catalyst of Fe–

Porphyrin-Based Metal–Organic Framework. *ACS Appl. Energy Mater.* **2018,** *1*, 4662-4669.

S9. Lu, C.; Yang, J.; Wei, S.; Bi, S.; Xia, Y.; Chen, M.; Hou, Y.; Qiu, M.; Yuan, C.; Su, Y.; Zhang, F.; Liang, H.; Zhuang, X., Atomic Ni Anchored Covalent Triazine Framework as High Efficient Electrocatalyst for Carbon Dioxide Conversion. *Adv. Funct. Mater.* **2019,** *29*, 1806884.
